# Teaching home-visitors to support responsive caregiving: A cluster randomized controlled trial of an online professional development program in Brazil

**DOI:** 10.7189/jogh.12.04007

**Published:** 2022-02-05

**Authors:** Nina Sokolovic, Alessandra Schneider, Michal Perlman, Rosângela Sousa, Jennifer M Jenkins

**Affiliations:** 1 Department of Applied Psychology and Human Development, Ontario Institute for Studies in Education, University of Toronto, Toronto, Ontario, Canada; 2 State Department of Social Assistance, Labour, and Human Rights, Piauí, Brazil

## Abstract

**Background:**

Home-visiting programs are a common and effective public health approach to promoting parent and child well-being, including in low- and middle-income countries. The World Health Organization and UNICEF have identified responsive caregiving as one key component of the nurturing care children need to survive and thrive. Nonetheless, the importance of responsive caregiving and how to coach it is often overlooked in trainings for staff in home-visiting programs.

**Methods:**

To determine whether it is possible to enhance home-visitors’ understanding of responsive caregiving and how to coach it, we conducted a cluster randomized controlled trial with 181 staff working in Brazil’s national home-visiting program. We used a computerized random number generator to randomly assign half of participants to take an online professional development course about responsive caregiving immediately and the other half to a waitlist. Individuals assessing outcome data were blind to group assignment.

**Results:**

Compared to those in the control group (N = 90, both randomized and analyzed), participants assigned to take the course (N = 91, both randomized and analyzed) were more knowledgeable about responsivity (Cohen’s *d* = 0.64, 95% Confidence Interval (CI) = 0.34, 0.94) and its importance for children’s socioemotional (odds ratio (OR) = 1.88, 95% CI = 1.00, 3.50) and cognitive (OR = 2.57, 95% CI = 1.15, 5.71) development, better able to identify responsive parental behaviors in videotaped interactions (*d* = 1.86, 95% CI = 1.51, 2.21), and suggested more effective strategies for coaching parents on responsivity (*d* = 0.51, 95% CI = 0.21, 0.80) and tracking goal implementation (OR* =* 3.20, 95% CI = 1.28, 7.99). There were no significant changes in participants’ tendency to encourage goal setting and reflection, or their perspective-taking skills. Participants were very satisfied with the course content and mode of delivery and there was no drop-out from the program.

**Conclusions:**

A short, online professional development program created moderate to large improvements in home-visitors’ knowledge and intended coaching practices. This suggests that such programs are feasible, even in low-income and rural areas, and provide a low-cost, scalable option for possibly maximizing the impact of home-visiting programs – particularly with regard to parental responsivity, and in turn, child outcomes.

Currently, 250 million children in low- and middle-income countries (LMICs) are failing to reach their developmental potential [1]. A popular approach to promoting healthy child development is through the use of home-visiting programs in which nurses or community health workers visit expecting and new mothers in their homes to provide knowledge and support as they transition to parenthood. A ground-breaking randomized trial of a home-visiting program in Jamaica demonstrated that children whose mothers received home visits had stronger motor and cognitive development in childhood [2], higher IQ and stronger language skills in adolescence [3], as well as less depression and violence, and 25% higher earnings, in adulthood [4,5]. The findings from this study have inspired similar programs to emerge across many LMICs, some of which have replicated impressive results for children’s cognitive, language, and motor development [6], while others have fallen short [7]. One explanation for these discrepancies is that the impact of home-visiting programs is closely related to the skills of home-visitors and the quality of service delivery [8]. In this study, we tested whether an online professional development program could be used to teach home-visitors in Brazil key information and skills needed to support responsive caregiving during home-visits.

Brazil is the largest country in South America with a population of over 200 million. In this upper middle-income country, one in eight children suffer from a psychiatric disorder [9] and one in three do not complete high school [10]. To improve health and education outcomes, in 2003 the federal government began enrolling the country’s lowest income families (monthly per capita earnings less than R$154, or approximately US$ 27) in a conditional cash-transfer program called Bolsa Família. In this program, families receive an average R$ 167, or approximately US$ 30 per month so long as mothers participate in prenatal care, their children follow the country’s vaccine schedule, and children maintain a minimum 85% school attendance [11]. In 2016, the government decided to launch a nationwide home-visiting program – *Criança Feliz* (CF) – to provide additional support for these vulnerable families. Families enrolled in CF receive hour-long visits once a month throughout a woman’s pregnancy and once a week through the first three years of a child’s life. During these visits, home-visitors are responsible for promoting parent-child play, conducting basic maternal and child health screenings, and referring families to services as needed. The long-term aims of this program are to improve maternal and child health, increase school completion rates, and reduce intergenerational poverty and violence in the country [12].

Achieving these ambitious goals is challenging, especially because we still know very little about how to make home visits most efficient and what works best for whom [13]. Nonetheless, the World Health Organization and UNICEF have produced a nurturing care framework which identifies five things children need during early development to survive and thrive, one of which is responsive caregiving [14]. Responsive caregiving is defined as a type of care in which caregivers recognizes children’s perspectives – their moods, needs, interests, and abilities – and provide consistent, timely, and appropriate responses. For example, this can involve recognizing that a child is upset because they are hungry and offering them food, noticing that a child has fixated on a certain object and asking them a question about that item of interest, and using developmentally appropriate language. Meta-analyses and randomized controlled trials have shown that responsive caregiving supports children’s language skills, cognitive abilities, and mental health [15-18]. Indeed, a Brazilian longitudinal cohort study found that the levels of responsive caregiving children received at age 4 were positively associated with their IQ at age 18 [19]. Moreover, according to a recent global meta-analysis of over 100 early intervention programs, programs with a focus on responsive caregiving tend to have a larger impact on children’s cognitive development than programs which do not [20].

Several studies demonstrate why enhancing responsive caregiving is particularly important in the Brazilian context. First, in Latino culture, parenting tends to emphasize direction and obedience rather than autonomy support and perspective-taking [21]; it has been demonstrated that children whose mothers provide more directives and fewer responsive expansions during interactions have poorer language scores [22]. Second, in 2012, a nationally representative survey in Brazil revealed that less than 20% of mothers viewed talking to children or playing with them as important for children’s early development [23]. Another Brazilian study reported that children of mothers who provided less cognitive stimulation were over three and a half times more likely to be developmentally delayed [24]. Importantly, there is evidence that parenting interventions in Brazil are effective. For example, one recent study found that parents from low-income regions in Brazil randomly assigned to a parenting program improved in their ability to engage in cognitively stimulating interactions, and their children made substantive gains in language and cognitive skills, compared to children whose parents did not participate in the intervention [25]. Recent randomized trials of two small, local home visiting programs in Brazil also found positive effects for the outcomes of mother-child attachment [26] and child development [27]. Hence, there is room for growth with respect to responsive caregiving in Brazil, and also reason to believe that such growth is possible.

The CF home-visiting program provides an excellent opportunity to enhance responsive caregiving in Brazil. However, to realize this potential, CF home-visitors need a deep understanding of what responsive parent-child interactions look like and how to coach parents to engage in more responsive interactions. Indeed, achieving impact in home visiting programs is dependent on having a skilled, well-trained, and well-supported workforce [28]; when home-visitors are responsive to families and can effectively facilitate parent-child interaction and promote parent and child engagement, the impacts of home-visiting programs on parent behavior and child outcomes tend to be larger [8]. The staff in Brazil’s CF program include high-school-educated home visitors who work with up to 30 families at any given time and university-educated municipal-level supervisors who oversee up to 15 home-visitors. All staff receive one 40-hour procedural training which covers the basics of home visits and one 40-hour training in UNICEF’s Care for Child Development (CCD) methodology. Even though CCD is intended to promote responsive caregiving [29], it emphasizes the quantity, and not necessarily quality, of parent-child interactions. For instance, CCD recommends “telling children the names of things and peoples” instead of labeling what children are looking at and already thinking about. Moreover, guidelines tell home-visitors to praise parents for talking or playing with their child, but do not mention praising parents for having conversations about what their children are interested in or letting children lead play interactions. Hence, even after completing the CCD training, home-visitors still have room to grow when it comes to confidently and consistently coaching parents to be more responsive caregivers [30].

Professional development (PD) programs offer one way to enhance home-visitors’ knowledge and skills. Indeed, research on training for early childhood development workers indicates that optimal outcomes are achieved when initial training is followed up by continued PD and support [31]. What should this PD look like? In a review of approaches to training staff implementing early learning interventions in LMICs, observational learning, flexibility, peer support, and high-quality supervision were all highlighted as important ingredients for program success [32]. In a review of trainings for home-visitors, primarily in high-income countries, key predictors of success included providing opportunities for home-visitors to observe models of, practice, get evaluated on, and reflect upon key skills, as well as spurring supervisors to provide ongoing praise and encouragement for home-visitors’ use of these skills [33]. Currently, most PD programs for home-visitors are delivered in the form of day-long, in-person training workshops [32]. However, this approach is costly, particularly in rural communities, and challenging to implement at scale. It is also not necessarily a wise investment when staff retention is low, as is the case in many early childhood programs in LMICs [32], including CF [12]. Online PD programs, on the other hand, offer a promising means of reaching many staff at a low cost [34,35]. To our knowledge, there is currently no research on online PD for home visitors. However, research on PD for early childhood educators and teachers has identified several core components of effective online learning: it is ongoing, covers content that is relevant and interesting to participants, and includes opportunities for social interaction and collaboration among participants [34].

Guided by these principles, we designed an online PD course about responsive caregiving – called Responsive Interactions for Learning (RIFL) – in Portuguese, Spanish, and English. To make the program easy to implement at a large scale, we designed it to be brief. However, given the documented importance of ongoing learning, peer support, and having supervisors who reinforce training content, we designed it to be taken simultaneously by both home-visitors and their supervisors. To encourage ongoing implementation, we also dedicated the last module of the course to making a plan for how supervisors and home-visitors would work together to implement the content covered in the course on an ongoing basis. In this study, we sought to evaluate whether online PD can be used to build the capacities of home-visiting staff in LMICs. Specifically, using a cluster randomized controlled trial, we wanted to assess whether participants who took an online PD course would have a better understanding of responsive caregiving and how to coach it compared to those on a waitlist.

## METHODS

### Recruitment and participants

Participants were CF home-visitors and supervisors working in Piauí – a predominantly rural and low-income state in Northeastern Brazil. Since the program operates via municipal hubs, program coordinators from the state government contacted municipalities and described the study and requirements for participation. Program staff who were interested in participating were invited to sign a consent form. While there were no exclusion criteria for staff, to be eligible to participate, a municipality had to obtain consent from at least one supervisor and one home-visitor. We aimed to recruit at least 50 municipalities based on reasonable power calculations for cluster randomized trials using an alpha of 0.05, beta of 0.2, and assuming an average of three participants per municipality, an intraclass correlation of 0.2, and an estimated effect size of 0.6 based on findings from other PD programs [33]. Ultimately, program coordinators enrolled 181 participants across 54 municipalities. There were 12 municipalities with two participants, 15 with three participants, 26 with four participants, and one with eight participants. Sample characteristics are presented in **Table 1**. Participants were compensated with a certificate of participation and souvenirs worth approximately US$10. All procedures were approved by the University of Toronto Research Ethics Board, including informed consent.

**Table 1:** Participant characteristics by group

	Intervention group (N = 91), N (%)	Control group (N = 90), N (%)	Difference test, *χ^2^* (*P*-value)
Female	81 (89%)	80 (89%)	<0.001 (0.98)
Supervisor	27 (30%)	28 (31%)	0.04 (0.83)
Work full-time (40h/week)	69 (78%)	74 (83%)	0.64 (0.42)
Prior training in home visiting	82 (90%)	76 (84%)	1.31 (0.25)
Age:			
<20 y	1 (1%)	1 (1%)	2.99 (0.39)
20-29 y	34 (37%)	26 (29%)	
30-39 y	34 (37%)	45 (50%)	
40+ years	22 (24%)	18 (20%)	
Education:			
High school diploma or less	28 (31%)	28 (31%)	1.09 (0.78)
Some university	13 (14%)	10 (11%)	
University diploma	37 (41%)	42 (47%)	
Post-university studies	13 (14%)	10 (11%)	
Experience working with children (years):			
<1	2 (2%)	4 (5%)	0.70 (0.70)
1-2	20 (24%)	19 (23%)	
3+	62 (74%)	61 (73%)	

### Randomization procedures

For this cluster randomized controlled trial we conducted a clustered, stratified randomization process. We randomized at the level of the municipality since staff working in the same municipality would be likely to share information relevant to the intervention with one another if randomized to different conditions. Municipalities were stratified based on two variables: (1) whether all staff had university-level education or not and (2) whether the majority of staff had completed their 80-hour home-visiting training. We used the combination of these two dichotomous variables to create four groups (high or low education, high or low previous training) and conducted randomization separately within each of these groups. A statistician who was not involved in the study used a computerized random number generator to randomly assign municipalities to the intervention or control condition with a 1:1 allocation ratio. The result was that 26 municipalities (91 individuals) were assigned to the intervention condition and 28 municipalities (90 individuals) were assigned to the control (ie, waitlist) condition. We did not inform staff of assignments until all baseline data collection was complete. As shown in **Table 1**, randomization was successful in that participant demographics did not differ systematically between the intervention and control groups.

### Responsive Interactions for Learning (RIFL) online course

The RIFL course was designed in alignment with best practices for home-visiting and online PD programs [33,34]. The course consisted of four modules. The key learning goals and example activities for each module are outlined in **Table 2**. Every module required approximately two and a half hours to complete, including both an asynchronous and synchronous component, as well as brief homework assignments. In the asynchronous component, participants viewed up to three lecture videos of less than 20min each, which included didactic teaching, video examples, and moments for participants to pause the video and write down their responses or reflections to a prompt posed by the lecturer. Following each lecture video, participants completed a brief multiple-choice quiz to check for understanding. Synchronous sessions lasted one and a half hours and were held on a video conferencing platform. A facilitator led participants through several discussion exercises and interactive activities, including watching and reflecting on videos of parent-child and home-visitor coaching interactions and subsequent role playing in which they practiced various scenarios. At the end of each module, participants completed an assignment that required them to put the skills taught in that module into practice (eg, practice having a responsive interaction, or coaching another person) and then write a brief reflection on the experience.

**Table 2:** Course outline: Learning goals and example activities

Learning goals	Example activities
Module 1: What are responsive interactions for learning?	
• Understands what responsive interactions looks like	• Compare and contrast videos of responsive vs unresponsive parent-child interactions
• Able to identify responsive vs unresponsive behaviors	• Pause videos of parent-child interactions to highlight specific (un)responsive behaviors
• Understands how responsive interactions support children’s development	• Discuss key findings from research about the benefits of responsivity
Module 2: Taking children’s perspectives	
• Able to watch a child and identify: (1) what mood a child is in; (2) where a child is looking; (3) what a child is thinking and; (4) the child’s developmental level (eg, motor skills, language)	• Watch videos of children playing and pause to analyze and explain children’s behavior
·	• Practice taking children’s perspective and commenting on what they might be looking at or thinking about during a home-visit
• Gives examples of parent behaviors that would help expand a child’s learning and development in a given moment	• Watch videos of parent-child interactions, identify missed opportunities, and offer suggestions for how to improve the quality of the interactions based on child’s interest/needs/developmental level
Module 3: Coaching parents	
• Understands the importance of being responsive to parents during home visits	• Compare and contrast videos of home-visitors who are responsive vs unresponsive to parents’ needs
• Knows effective coaching strategies (ie, praise, broadcasting, modeling)	• Watch videos of home-visiting interactions and offer suggestions for how to coach the parent to have more responsive interactions • Role play to practice using coaching strategies
• Understands the importance of involving other family members so that they too learn to be more responsive	• Discuss and practice using strategies for involving other family members (eg, siblings, fathers) in home visits
Module 4: Working as a team	
• Helps parents set and track goals	• Practice setting goals based on video examples and role plays
• Understands the importance of being responsive to colleagues	• Compare and contrast videos of responsive vs unresponsive supervisors
• Supports colleagues in implementing course lessons	• Define shared goals for the team and make a plan for implementation and tracking

For this study, the online course was administered over an eight-week period between October and December 2019. Participants had two weeks to complete each module: one week to complete the asynchronous material, and the following week to attend a synchronous session. Participants were divided into five sections, each with up to 18 participants. A separate synchronous session was hosted for each section, though all sessions were facilitated by the same person (a course and study author, AS). To ensure fidelity, the facilitator completed a checklist after each session to monitor whether the same content was covered in all sections. Since most participants did not have internet access at home, they completed both the asynchronous and synchronous components of the course in their municipal offices. Time spent on the course was included in their working hours. To promote community-building, participants were encouraged to watch the online lectures with their colleagues, though they were required to complete quizzes and assignments independently. Furthermore, all participants were invited to join a group messaging app with other participants in their assigned section. Participants in the control group did not receive any PD beyond the 80-hour CF training described above. They were offered the opportunity to take the RIFL course after the end of the evaluation.

### Measures

**Recognizing and coaching responsivity.** At the end of the study, trained Portuguese-speaking research assistants who were blind to participant condition conducted 30-minute structured interviews with participants on a video-conferencing platform to assess their understanding of responsivity and coaching. Participants were asked to: (1) describe the benefits of responsive interactions for children’s development, (2) watch two videos of parent-child interactions and identify what the parent did well and what the parent could improve in each video, (3) explain what strategies they would use to coach the parent in one of the videos they watched, and (4) explain how they would support the parent to practice and implement suggestions. For each interview question, we pre-specified correct responses (eg, a correct response to “what could this mother have done better?” would including any statement about noticing the child’s interests and following the child’s lead). Standard probes were used to help participants focus their answers toward the interest of the investigator (ie, the pre-specified correct responses). The interview guide is available in the online supplementary materials.

All interviews were video-recorded and later scored by trained Portuguese-speaking coders who were blind to participant condition. Codes were assigned as follows: 0 = no appropriate response, 1 = vague or incomplete response, 2 = complete and accurate response. Coders scored the quality of participants’ responses both before and after the interviewer provided the standard probes.

Responses for two of the four interview questions had good internal consistency: α = 0.73 for identifying (un)responsive behaviors in videos of interactions (ten items); α = 0.73 for coaching strategies (three items). Since the responses for the other questions had consistencies below 0.7, we analyzed individual items separately. For the benefits of responsivity, these items were identifying (1) socioemotional and (2) cognitive benefits. For supporting implementation, these items were (1) setting goals, (2) tracking goal implementation, and (3) monitoring progress.

To ensure reliability, 20% of videos were double coded by an expert coder. The interrater reliabilities, evaluated using Cronbach’s alphas [36], were 0.97 for evaluating videos; 0.95 for coaching strategies, and ranged from 0.71 to 0.82 for the individual items assessing participants understanding of why responsivity matters for child development and how to monitor parent progress.

**Knowledge of responsivity.** In addition to the interview, at the beginning and end of the study, participants completed a knowledge test online. This test was developed by the study authors to assess participants’ knowledge of responsivity, as no such knowledge test currently exists in the literature. For this questionnaire, participants read a scenario describing a play interaction between a mother and child building a block castle together. They were then asked to assess the extent to which each of 12 maternal actions would promote the child’s learning and development on a five-point Likert scale ranging from zero (not at all) to five (a great deal). Scores were calculated as the mean of items, wherein unresponsive actions were reverse-coded.

Before its use in this study, the scale went through an iterative process of piloting and revisions with Canadian students in a teacher education program. Exploratory factor analysis revealed a two-factor model, which was replicated in this study. However, the internal consistency of one factor was low (0.45 at pre-test, 0.68 at post-test) in this study, so it was dropped from analyses. The second factor, which consists entirely of reverse-coded items and reflects the importance of following children’s lead, has seven items (eg, “discourage the child if he starts building a boat instead of a castle”, reverse-coded). The internal consistency of this factor in this study was 0.74 at pre-test and 0.71 at post-test.

**Perspective-taking ability.** The RIFL course requires participants to practice understanding both children’s and mothers’ perspectives. We assessed participants’ perspective taking abilities in this study to determine whether taking this course increased participants’ ability to take other people’s perspective in their daily life, or whether participants with lower initial perspective-taking abilities had more to gain from participating in this course. Participants completed the Perspective Taking subscale of the self-reported Interpersonal Reactivity Index [37] pre- and post-intervention. This widely used scale has been translated into Portuguese and validated in a Brazilian sample [38]. The perspective-taking sub-scale has seven items (eg, “when I'm upset at someone, I usually try to “put myself in his shoes” for a while”) each rated on a five-point Likert scale.

**Course satisfaction.** Participants who took the course were asked to rate their satisfaction with the course using an online questionnaire. They rated the extent to which they enjoyed the various course components, how much they felt they learnt from the course, and how strongly they would recommend it (see Table S1 in the **Online Supplementary Document** for items). Each statement was rated on a Likert scale from one (disagree strongly) to five (agree strongly). In addition, we asked participants two open-ended questions: “What did you enjoy most about your experience in this course?” and “What did you enjoy least?”

**Demographics.** At the beginning of the intervention, all participants completed an online demographic questionnaire reporting on their employment, age, highest educational attainment, and how many years of experience they have working with children.

### Statistical analysis

We conducted descriptive analyses for all outcome measures to inform model-building. Since individual interview responses were measured on an ordinal scale, we used Spearman correlations to test the correlations between before- and after-probe scores. Since they were all significantly correlated (ρ *=* 0.25-0.88, *P* < 0.01), and the average score was less than one for nearly all responses, we calculated scores using the maximum of the before- and after-probe scores to minimize the chance of floor effects. However, we also made the decision to conduct sensitivity analyses testing whether using the before- or after-probe scores changed our results.

We then used variance partitioning analysis to calculate intraclass correlation coefficients for all outcomes. As all intraclass correlation coefficients were significant, we used multilevel modeling to account for the clustering of participants in municipalities.

We built ordinal logistic multilevel mixed-effects regression models for the interview responses that were not combined (socioemotional benefits, cognitive benefits, goal setting, goal tracking, and monitoring progress) and multilevel mixed-effects linear regression models for continuous outcomes (identifying (un)responsive behaviors and coaching responsivity from the interview, as well as the knowledge test and perspective-taking measure). Since the intervention and control groups did not differ along any demographic variable, no demographic covariates were included in the models. However, for the outcomes for which we had pre-tests (knowledge and perspective-taking), we used pre-scores as covariates. For ordinal outcomes we reported Odds Ratios while for continuous outcomes, we calculated standardized effects sizes (Cohen’s *d*) by standardizing the coefficient using the variable’s pooled standard deviation [39]. Since we tested nine distinct outcomes, we used the Benjamini-Hochberg method [40] to calculate BH-adjusted *p*-values (*p*^BH^) that provide a more conservative estimate for assessing statistical significance against the standard 5% cutoff.

To further examine what works and for whom, we tested whether outcomes were associated with course attendance for those in the intervention group. Next, we used interaction terms to test whether intervention effects were moderated by key demographic characteristics for which there was sufficient variation in the sample. Because of their distributions (see **Table 1**), variables were dichotomized as follows: whether participants were a supervisor ( = 0) or home-visitor ( = 1), their age (<30 = 0, ≥30 = 1), their level of education (less than a university degree = 0, university degree or higher = 1), and their experience working with children (<3 years = 0, 3+ years = 1). Finally, we assessed whether participants who had stronger perspective taking showed more or less improvement in their understanding of responsivity and how to coach it. All statistical analyses were conducted in Stata v15.1 (StataCorp, College Station TX, USA) using multiple imputation to handle missing data. Qualitative data were analyzed using thematic analysis [41].

## RESULTS

### Implementation

There was no drop-out from the study: all participants (N = 181) completed both pre- and post-course online questionnaires, and all participants assigned to take the course completed it. Completion of the asynchronous elements of the online course was high: participants completed 81% of quizzes and 70% of assignments. The average accuracy on the quizzes was 83%, indicating strong comprehension of material presented in the online lecture videos. Average attendance at the synchronous sessions was also very high (88%). The primary reasons for non-attendance were unpredictable internet disconnection and individual absences due to illness or family obligations. Fidelity of synchronous sessions was 100%, as all pre-specified activities were completed for all modules and sections. Of the 181 enrolled participants, 171 completed the post-course interview. Reasons for non-completion of the interview included scheduling constraints and internet outages.

### Intervention effects

**Figure 1:**
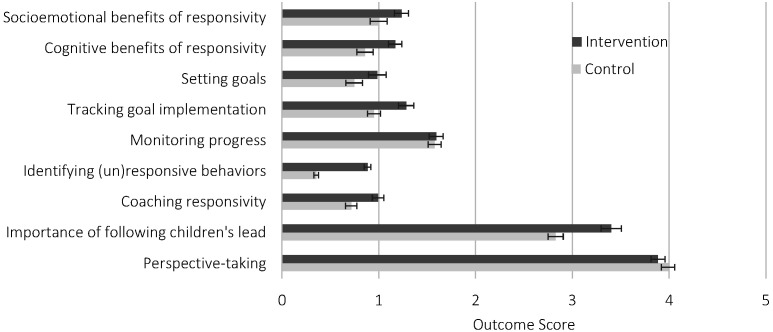
Post-course mean scores by intervention group with standard errors. The first seven outcomes (from the interview) were rated on scales with a maximum of 2; the last two outcomes were rated on 5-point Likert scales.

The impacts of the intervention are displayed in **Figure 1**. As detailed in **Table 3**, participants who took the course were over two and a half times more likely to give a more complete and accurate description of how responsive caregiving supports children’s cognitive development (ie, 2.6 times more likely to score a one or a two compared to a zero, or to score a two compared to a one or zero) and were also approximately twice as likely to provide a better description of the socioemotional benefits of responsivity (although this effect was only marginally significant when considering corrected *p*-values). Further, participants who took the course were over three times as likely to suggest more effective methods for supporting mothers to practice and track the implementation of goals between visits. They were also better able to identify responsive and unresponsive behaviors in videos of mother-child interactions (large effect size), and when asked how they would work with a mother to improve her responsivity, participants who had taken the course offered more effective coaching strategies (moderate effect size). On the knowledge test, participants who took the course were significantly better able to recognize that following children’s lead is important for children’s development, controlling for their pre-intervention knowledge (moderate effect size). The intervention did not impact the extent to which staff suggested setting goals or checking in at the start of each session to monitor goal implementation, or participants’ perspective-taking skills. Results showed the same pattern across outcomes when we used scores of before-probe or after-probe interview responses, instead of the maximum of the two.

**Table 3:** Intervention effects*

	Effect size	95% confidence interval	*P*-value	Adjusted *P*-value (*p*^BH^)
**Ordinal outcomes:**				
Socioemotional benefits of responsivity	1.88	(1.00, 3.50)	0.05	0.07
Cognitive benefits of responsivity	2.57	(1.15, 5.71)	0.02	0.04
Setting goals	1.80	(0.87, 3.69)	0.11	0.14
Tracking goal implementation	3.20	(1.28, 7.99)	0.01	0.04
Monitoring progress	0.95	(0.47, 1.92)	0.88	0.88
**Continuous outcomes:**				
Identifying (un) responsive behaviors	1.86	(1.51, 2.21)	<0.001	0.004
Coaching responsivity	0.51	(0.21, 0.80)	0.02	
Importance of following children’s lead	0.64	(0.34, 0.94)	<0.001	0.009
Perspective taking	-0.03	(-0.32, 0.27)	0.85	0.95

*Effect size is odds ratio (OR) for ordinal outcomes and Cohen’s *d* for continuous outcomes.

### Correlates of outcomes scores and moderator analyses

Certain outcome scores were correlated with participants’ course attendance and performance (see Table S2 in the **Online Supplementary Document**). Specifically, individuals who offered better suggestions for how to track goal implementation, on average, had attended more interactive sessions (ρ = 0.24, *P* = 0.04) and participants who were better able to identify the importance of following children’s lead in interactions had completed more quizzes (*r* = 0.21, *P* = 0.04). The intervention effects did not differ according to participant role, age, education, experience with children, or perspective-taking abilities (see Table S3 in the **Online Supplementary Document**).

### Course satisfaction

Satisfaction with all components of the course was high (see Table S1 in the **Online Supplementary Document**). On a five-point scale, participants thought the content of the course was novel (mean (M) = 3.88, standard deviation (SD) = 0.96), relevant to their work (M = 4.57, SD = 0.75), and compatible with the CF program (M = 4.66, SD = 0.64). They rated all aspects of the course (eg, lecture videos, assignments, synchronous sessions) as valuable to their learning (M = 4.42 to 4.60). They felt strongly that working in teams within their municipalities helped their learning (M = 4.79, SD = 0.41), they were confident in their ability to use what they had learned (M = 4.57, SD = 0.44), and believed that their participation in the course would make them better able to support families (M = 4.66, SD = 0.67). Many also agreed that the course changed the way that they interacted with children in their own lives (M = 4.47, SD = 0.74). Overall satisfaction was very high (M = 4.69, SD = 0.46) and all participants would recommend the course to others (M = 4.78, SD = 0.42).

There was also strong use of the group messaging platforms. Participants used these groups to share photos of themselves working on the course content with their colleagues and to send encouraging messages to others in different municipalities. Several themes emerged from participants’ responses to the question “What did you enjoy most about your experience in this course?”. The most common theme was that participants really enjoyed working with their peers (n = 33); for example, they highlighted “opportunities to interact with other municipalities” and “being able to role play and share cases with my colleagues.” Many participants also highlighted the emphasis on understanding children’s perspectives (n = 12), noting “I learned that we should always value children’s interests and ideas” and that I “learned to listen, observe, and praise more.” Other common responses included learning more about the concept of responsivity (n = 12), the focus on what to teach caregivers and how (n = 10), the META questions acronym (n = 7), and the use of video examples (n = 6). With regards to what individuals didn’t like, the most common answers included that the course was too short/condensed (n = 19) and technological challenges with the online course platform (n = 8) or video conferencing software (n = 7).

## DISCUSSION

Home-visiting programs are a promising approach to ensuring children in LMICs receive the nurturing care they need to reach their developmental potential [35]. However, there are limited data and no clear consensus on how to best train and support staff in LMICs to implement responsive caregiving and early learning interventions, such as home-visiting programs, especially at a large scale [32]. In this study, we took one step towards narrowing that knowledge gap by testing the use of online PD course for staff in home-visiting programs. Results demonstrate that a 12-hour online course can be used to build the knowledge and capacities of home-visiting staff, including those working in remote regions.

Specifically, this training, which focuses on responsive caregiving, increased home-visitors’ understanding of responsive caregiving, its importance, and how to effectively coach parents to engage in more responsive interactions with their children. The largest effect was seen for participants’ ability to identify responsive behaviors and missed opportunities in videos of parent-child interactions. This was unsurprising, given that this skill was practiced in almost all components of the course. Indeed, most asynchronous lectures included videos of parent-child interactions with interpretations, several assignments required participants to analyze or reflect on videos of parent-child interactions, and all synchronous sessions required participants to complete discussions or role plays based on videos of parent-child interactions. We believe this skill is foundational for coaching responsive caregiving, which requires knowing which behaviors to praise and which behaviors to coach during home-visits. The effect sizes for most other outcomes – including understanding what responsive behaviors are, how they support child development, and what strategies to use to coach responsivity – were moderate. These are in line with findings from other adult training programs [33].

Although the course improved some intended coaching practices, participants who took the course were not significantly more likely than those on the waitlist to suggest setting goals and checking in about progress as means to encourage parents to practice responsivity skills in between home-visits. Given that these are important means of promoting behavior change, these topics will receive greater emphasis in future iterations of the course. The course also did not impact participants’ perspective-taking skills. Although perspective taking has been shown to be highly stable in adults [42,43], based on previous training studies we did hypothesize improvement [44]. We may not have seen this because the emphasis in this course was not on building home-visitors’ perspective-taking skills, but rather equipping them to be able to build parents’ perspective-taking skills. Notably, learning outcomes did not differ based on participant demographics. There was also limited evidence that course engagement (ie, quiz completion, synchronous session attendance) was associated with learning outcomes.

A remarkable result of this efficacy study was that there was a 100% completion rate and extremely high attendance. This was attributable to a strong commitment from the state government and program coordinators. Participants completed the course during their workday, a key indicator of the government’s support of this program. Furthermore, state government and program coordinators provided technical support to municipalities and sent reminders to reinforce participants’ participation. This support was essential because many participants had low levels of technological literacy and several municipalities encountered intermittent internet connectivity problems, which forced them to miss synchronous sessions or made them unable to view lecture videos. The fact that participants were willing to learn skills related to operating online courses and video conferencing platforms, rearrange their schedules to attend other synchronous sessions when needed, and persevere to complete the course is a testament to the efforts of the program coordinators and the commitment and desire of participants to develop their professional skills and expertise. Indeed, many participants expressed that they wished the course had been longer or covered more. However, it remains to be seen whether this can be replicated at scale and what key ingredients contribute to course engagement and impact.

The high levels of engagement in this course may be explained by the strong learning communities that were built in the program. Participants formed communities within and across municipalities while working through the asynchronous content together, during discussions and exercises in the synchronous sessions, and on the group messaging platforms created for each section. Participants reported strong satisfaction with the team-based learning approach, and noted this as one of the things they enjoyed most about this course, reaffirming past findings that peer interaction is a valuable aspect of staff training [32] and that effective online PD should include opportunities for social interaction and collaboration [34]. Another factor that may have contributed to participant motivation and engagements was that participants found the course to be relevant and important to their day-to-day work. Past research suggests that this is a key ingredient of effective online trainings [34]. Altogether, the high engagement and strong reported satisfaction in this course indicate that administering PD courses to home-visitors online is a feasible approach when the content and mode of delivery are designed in alignment with what we know about effective PD and online learning.

In designing this program, several steps were taken to partner with the national government and plan for scale. One of our team members was Brazilian and had longstanding connections with persons in the Federal Ministry of Citizenship responsible for implementing the CF program. This helped us to understand the program’s needs, and possibilities for this course, which were used to inform the project proposal. Once funding was secured for the project, a memorandum of cooperation was signed with the Ministry to formalize the cooperation. Thereafter, regular meetings took place to present updates, collect feedback, and adapt accordingly. This procedure ensured that the final product was something which the Ministry found to be valuable and feasible for national implementation. The intention is that in the years to come this course will become integrated into the mandatory training for staff in the CF program nationwide.

### Limitations and future directions

The results of this study should be considered in light of several limitations. First, we were only able to assess participants’ knowledge and intended coaching practices. Furthermore, we had to develop measures to do so, as no existing measures assessed the constructs targeted in this course. Although these measures showed good reliability, it is hard to interpret effects with new measures. For this reason, it will be important to develop standardized measures of these skills that make it is easier to translate improvements into observed impacts. In the future, we also hope to collect observational data in homes to assess the impact of this course on home visitor’s coaching behaviours, maternal responsivity, and ultimately on child development. In addition, although the RIFL course can be delivered entirely online and thus completed remotely, in its present form it requires a facilitator to lead the synchronous component. To increase scalability, it would be valuable to develop a version of the course that can be delivered in an asynchronous format and test its effectiveness. This would make it possible to administer this course at almost no cost beyond participants’ time, making it an attractive option for large-scale programs. Given that countries around the world are increasingly recognizing the value of investing in responsive caregiving, it will also be valuable to test whether this program can be implemented effectively in different regions of the world. The existing Portuguese, Spanish, and English versions use culturally-tailored video examples but further research is needed to understand to what extent this promotes understanding and engagement and what, if any, cultural adaptations are needed to replicate positive results in other contexts.

## CONCLUSIONS

The results of this efficacy study demonstrate that online learning is a viable approach to capacity-building in home-visiting programs, including in rural and low-income regions of LMICs with high-school educated staff. Specifically, in this context we found that offering the RIFL course to staff in home-visiting programs increased the ability of home-visitors to identify responsive parental behaviors and work with parents effectively to increase the frequency of responsive caregiving. By targeting this critical component of nurturing care, home-visiting programs may be able to maximize their impact on children’s development. Indeed, if results can be replicated at scale, online PD could increase the potential of home-visiting programs to generate substantial benefits and cost savings to society, at a minimal added cost.

## Additional material


Online Supplementary Document
