# Supplementary material for: Teaching home-visitors to support responsive caregiving: A cluster randomized controlled trial of an online professional development program in Brazil
Source: J Glob Health. 2022 Feb 5;12:04007. doi: 10.7189/jogh.12.04007 (PMC8818297; doi:10.7189/jogh.12.04007)
Supplement: Online Supplementary Document [file jogh-12-04007-s001.pdf]

Promoting Responsive Caregiving in Brazil: A Cluster Randomized Controlled Trial of an Online Professional Development Course for Home-Visitors

Supplemental Materials

Interview Guide

Q1. WHY IS RESPONSIVITY IMPORTANT

Tell me about why you think it's important to promote good parenting interactions.

Expected response #1: Good interactions help support children's socio-emotional development (i.e., mental health/confidence/attachment security).

Probe: How do you think it might make a difference to how children behave?

Expected response #2: Good interactions help to promote children's language and cognitive development

Probe: How do you think it might make a difference to children's learning?

Q2. INDENTIFYING (UN)RESPONSIVE BEHAVIORS

Video 1, Part 1. Tell me a little bit about what you noticed about how the mother and child interacted together. Please tell me briefly what this mother did very well.

Expected response #1: The mother demonstrated an awareness of and sensitivity toward the child's mood (happy) AND interests (toys)

Probe: Did you get the sense that the mother understood the child's thoughts?

Expected response #2: The mother did a good job following the child's interest (cooking toys) and not being too directive or taking over the interaction

Probe: What did the mother do to keep the child engaged?

Expected response #3: The mother used diverse language and questions which can help support child's vocabulary growth and learning.

Probe: Can you comment on the language the mother was using?

Video 1, Part 2. Next, can you tell me about what the mother could have done differently to have an even better interaction?

Expected response #4: The mother talked too much and should have done more watching/waiting to support a better back-and-forth interaction.

Probe: Is there anything the mother could have done differently to be more responsive to the child's mood, wants, or interests?

Expected response #5: The mother didn't work in the child's zone of proximal development (i.e., "learning zone") or add on to the interaction in a way that would create opportunities for the child to practice/learn language or motor skills.

Probe: Is there anything the mother could have done differently to better support the child's learning and development?

Video 2, Part 1. Tell me a little bit about what you noticed about how the mother and child interacted together. Please tell me briefly what this mother did very well.

Expected response #6: The mother used diverse language and questions which can help support child's vocabulary growth and learning.

Probe: Can you comment on the language the mother was using?

Video 2, Part 2. Next, can you tell me about what the mother could have done differently to have an even better interaction?

Expected response #7: The mother was not following the child's lead and just doing what she thought would be interesting with the toys (playing by herself) and telling the child what to do

Probe: Is there anything the mother could have done differently to be more responsive to the child's mood, wants, or interests?

Expected response #8: The mother talked too much and should have done more watching/waiting to support a better back-and-forth interaction.

Probe: How else could she have been more responsive to the child?

Expected response #9: The mother didn't work in the child's zone of proximal development (i.e., "learning zone") or add on to the interaction in a way that would create opportunities for the child to practice/learn language or motor skills.

Probe: Is there anything the mother could have done differently to better support the child's learning and development?

Expected response #10: The mother could have been more sensitive/responsive to the child and his perspective (i.e., wants/interests/ability levels).

Probe: What would you have done to improve the quality of the interaction?

Q3. HOW TO COACH RESPONSIVITY

Can you pick one of the things that you would change from the previous question and tell me, in the role of a home visitor, what you would do or say to help this mother make this change?

Expected response #1: Either praise the mother or broadcast the mother's actions, with detail about how/when to use this strategy and/or why it works.

Probe: What might you, as a home visitor, do or say to help the mother realize what she was doing well?

Expected response #2: Either model a responsive behavior for the mother or broadcast the child's perspective, with detail about how/when to use this strategy and/or why it works.

Probe: What might you, as a home visitor, do or say to help the mother realize what she was not doing as well?

Expected response #3: Names several effective coaching strategies (e.g., praise, model, broadcast parent actions, broadcast child perspective).

Probe: What strategies would you use to coach the mother?

Q4. HOW TO PROMOTE AND MONITOR PARENT PROGRESS

You have just told me what you would help the mother to change her behavior. Now can you tell me, how would you ensure this change is occurring?

Expected response #1: Work with the mother to set a clear and explicit goal at the end of each visit.

Probe: How would you help the mother know what to work on?

Expected response #2: Encourage the mother to make a system to keep track of her own practice/progress on the goal (e.g., check marks on a calendar indicating days she worked on the goal).

Probe: How would you encourage the mother to practice?

Expected response #3: Start the next visit by asking about how practice of the goal set during the previous visit went.

Probe: How would you know whether the mother is using your suggestions?

Table S12022-02-04 7:05:00 PM

Course Satisfaction rated on 5-point Likert scales

**Satisfaction with Course Components**	Mean (SD)
Online lectures	4.58 (.50)
Video examples	4.60 (.49)
Pauses to write and reflect	4.42 (.60)
Online quizzes	4.47 (.62)
Online assignments	4.56 (.62)
Discussions during synchronous sessions	4.59 (.61)
Activities during synchronous sessions	4.47 (.69)
**Satisfaction with Overall Course**	
It was helpful to my learning to take the course in a team with my colleagues.	4.79 (.41)
What I learned in this course is new or different from what I already knew.	3.88 (.96)
What I learnt in this course is relevant to me and my work as a supervisor/home-visitor.	4.57 (.75)
What I learnt in this course is compatible with the Crianca Feliz program.	4.66 (.64)
I am confident that I understand what makes for a responsive interaction for learning.	4.59 (.49)
I am confident that I will be able to use what I have learnt to support families to have more responsive interactions for learning.	4.75 (.44)
After having participated in this course, I have changed / will change how I interact with children in my personal life (as a parent, aunt, grandparent, …).	4.47 (.74)
Having participated in this course has made me / will make me better able to support families and child development.	4.66 (.67)
I have shared / plan to share what I have learnt with other supervisors/home-visitors.	4.48 (.66)
I would recommend this course to other supervisors/home-visitors.	4.78 (.42)
I am satisfied with my experience in the course.	4.69 (.46)

Table S2

Correlations between outcomes and course attendance/performance in the intervention group

	% of Quizzes Completed	Average Accuracy on Quizzes	% of Assignments Completed	Attendance at Synchronous Sessions
Socioemotional benefits of responsivity	-0.07 (0.57)	0.02 (0.84)	-0.05 (0.66)	0.17 (0.14)
Cognitive benefits of responsivity	0.08 (0.50)	0.15 (0.22)	0.04 (0.73)	0.02 (0.88)
Identifying (un)responsive behaviors	-0.02 (0.86)	0.12 (0.28)	0.02 (0.83)	0.09 (0.40)
Coaching responsivity	-0.04 (0.71)	0.17 (0.12)	0.10 (0.36)	-0.08 (0.46)
Setting goals	-0.15 (0.20)	0.13 (0.29)	-0.07 (0.57)	0.05 (0.69)
Tracking goal implementation	-0.09 (0.47)	0.29 (0.02)*	0.09 (0.45)	0.23 (0.04)*
Monitoring progress	-0.14 (0.23)	0.20 (0.09)	-0.07 (0.57)	-0.01 (0.94)
Following children's lead	0.21 (0.04)*	0.05 (0.66)	0.15 (0.17)	0.18 (0.08)
Perspective-Taking	-0.09 (0.42)	0.20 (0.08)	0.07 (0.49)	-0.06 (0.59)

Values represent Pearson correlations (*r*) followed by two-tailed *p*-values in parentheses.

Table S3

Moderation of the intervention effects

	Supervisor vs. Home-visitor	Age (<30 years vs. 30+ years)	Education (<university degree vs. university degree +)	Experience working with kids (<3 years vs. 3+ years)	Pre-Intervention Perspective-Taking
Socioemotional benefits of responsivity	-0.28 (0.29)	0.28 (0.30)	0.02 (0.94)	-0.26 (0.32)	-0.01 (0.94)
Cognitive benefits of responsivity	-0.04 (0.87)	0.29 (0.23)	0.27 (0.21)	0.26 (0.27)	0.13 (0.52)
Identifying (un)responsive behaviors	-0.05 (0.58)	0.10 (0.25)	0.14 (0.08)	-0.07 (0.49)	-0.02 (0.78)
Coaching responsivity	0.001 (0.99)	0.05 (0.77)	0.22 (0.18)	-0.28 (0.13)	0.06 (0.68)
Setting goals	-0.24 (0.37)	0.15 (0.60)	-0.12 (0.66)	-0.18 (0.53)	0.09 (0.68)
Tracking goal implementation	-0.007 (0.98)	0.37 (0.09)	0.25 (0.23)	0.28 (0.24)	0.29 (0.10)
Monitoring progress	-0.07 (0.76)	-0.18 (0.38)	0.09 (0.64)	0.10 (0.67)	0.14 (0.39)
Following children’s lead	0.10 (0.70)	-0.08 (0.75)	0.17 (0.46)	-0.13 (0.64)	0.06 (0.76)
Perspective-Taking	0.06 (0.74)	0.0006 (0.99)	0.11 (0.53)	-0.20 (0.34)	N/A

Values represent regression coefficients (b) for interaction terms followed by two-tailed *p*-values in parentheses.
